# Application of stem cell-derived exosomes in skin wound healing: mechanisms, prospects, and challenges

**DOI:** 10.3389/fimmu.2026.1846916

**Published:** 2026-08-25

**Authors:** Yihang Li, Fuhong Zheng, Yajuan Bai, Yang Yu, Ran Zhang, Qiang Feng, Yingqing Yu, Lei Zhu, Dongxu Wang, Haibo Liu

**Affiliations:** 1 Department of Emergency, The First Hospital of Jilin University, Changchun, Jilin, China; 2 Laboratory Animal Center, College of Animal Science, Jilin University, Changchun, China

**Keywords:** exosomes, extracellular vesicles, mesenchymal stem cells, plant-derived exosome-like nanovesicles, wound healing

## Abstract

As the largest organ of the human body, the skin is essential for maintaining systemic homeostasis; however, it remains highly susceptible to injury and wound formation, posing a significant clinical and economic burden. Wound healing is a complex, tightly coordinated process that progresses through overlapping phases of hemostasis, inflammation, proliferation, and tissue remodeling. Exosomes, nanoscale extracellular vesicles (50–150 nm in diameter) secreted by various cell types, have emerged as critical mediators of intercellular communication. Laden with bioactive molecules such as proteins, RNAs, and lipids, exosomes can partially replicate the regenerative functions of their parent cells. Among various types of exosomes, mesenchymal stem cell-derived exosomes (MSC-Exos) have become the most clinically translatable research direction in the field of skin wound repair due to their biological functions similar to those of their parent stem cells, low immunogenicity, and advantages as a cell-free therapy. This review focuses on MSC-Exos and systematically summarizes the roles and underlying mechanisms of adipose-derived stem cell exosomes, bone marrow mesenchymal stem cell exosomes, human umbilical cord mesenchymal stem cell exosomes, antler stem cell exosomes, human urine-derived stem cell exosomes, and induced pluripotent stem cell exosomes in cutaneous wound repair. Additionally, to broaden the diversity of therapeutic sources, plant-derived exosome-like nanovesicles are also discussed as an emerging perspective. Current evidence indicates that these vesicles facilitate wound healing and attenuate scarring by modulating multiple signaling pathways, thereby promoting cell proliferation, migration, angiogenesis, and extracellular matrix remodeling. Nevertheless, challenges in isolation, purification, optimization, standardization, and quality control continue to hinder their broad clinical translation. Despite these obstacles, exosomes represent a promising therapeutic strategy for cutaneous wound healing.

## 1 Introduction

The skin, the largest organ of the human body, constitutes approximately 15% of total body weight (1). Structurally and functionally intact skin is essential for maintaining internal homeostasis (2), serving as a mechanical barrier, preventing excessive fluid loss, and playing critical roles in thermoregulation, sensory perception, and immune regulation (3). Nevertheless, both internal pathological conditions and external mechanical injuries can disrupt skin integrity, leading to wound formation (4). Such wounds may induce a cascade of local and systemic pathophysiological responses, ultimately posing considerable challenges to patients and healthcare systems alike (5). Historically, wound management has remained a persistent clinical difficulty (6) and continues to impose a substantial burden on healthcare infrastructure (7), with significant socioeconomic repercussions (8, 9). Annual healthcare costs for acute and chronic wound care are estimated between US$28.1 and 96.8 billion, while the global wound care products market is projected to reach US$15–22 billion by 2024 (10). Therefore, the exploration and development of innovative therapeutic strategies for skin wound repair represent an urgent medical priority worldwide (8, 11, 12).

Wound healing is a vital and complex physiological process that involves the coordinated activity of keratinocytes, fibroblasts, vascular endothelial cells, recruited immune cells, and the extracellular matrix (ECM). This process entails the replacement, repair, and reconstruction of lost cellular structures and tissue architecture through the synchronized interplay of multiple cell types and tissues (13). Skin wound healing is a temporally overlapping and interdependent continuum, classically described in four sequential phases: hemostasis, inflammation, proliferation, and remodeling (14–16). The reconstruction of both the epidermal and dermal layers is governed by numerous factors, and impaired or delayed healing can lead to significant clinical complications (17). In the complex and finely orchestrated process of skin wound repair, efficient intercellular communication and signal transduction are central to the orderly progression of each phase. Recent studies have revealed that exosomes (Exos) serve as the key messengers that mediate this intercellular communication and coordinate the entire wound healing process.

Exosomes are small vesicles that are secreted by cells, enclosed by a lipid bilayer membrane, and range from 50 to 150 nm in size (18, 19). Exosomes are widely distributed in fluids such as urine, blood, serum, breast milk, amniotic fluid, cerebrospinal fluid, malignant ascites, saliva, bile, and lymph, and they have been detected in nearly all body fluids under both healthy and diseased conditions (20–25). Initially, exosomes were considered cellular debris or waste products and were thought to indicate cell death (26, 27). However, subsequent extensive studies have demonstrated that exosomes are crucial mediators of extracellular signal transduction (28). Exosomes contain a variety of components, including proteins, mRNAs, microRNAs (miRNAs), long noncoding RNAs (lncRNAs), and lipids. Due to their specialized structure, they can enter cells via endocytosis and play a critical role in intercellular communication (18, 19, 29–32). The primary database utilized for exosome research is ExoCarta (available at http://www.exocarta.org, accessed on 16 July 2025). The current version of ExoCarta catalogues 119,489 proteins, over 19,000 RNA entries, and 3,946 lipid molecules, compiled from 1,249 exosomal studies.

Exosomes function both as cellular products and as functional reflections of their parental cells. Consequently, stem cell-derived exosomes exhibit biological activities analogous to those of the stem cells themselves. These vesicles are regarded as key mediators of paracrine signaling and represent a promising platform for developing cell-free therapeutic strategies (33). For instance, mesenchymal stem cell-derived exosomes (MSC-Exos) carry cytokines such as vascular endothelial growth factor (VEGF), transforming growth factor-β1 (TGF-β1), interleukin-6 (IL-6), IL-10, and hepatocyte growth factor (HGF), which collectively promote angiogenesis and immunomodulation (34). Research over recent decades has demonstrated the therapeutic potential of MSC-Exos across a wide range of conditions, including neurological, respiratory, cartilage, renal, cardiac, and hepatic diseases, as well as in bone repair and cancer (35–44). Preclinical studies have shown that MSC-Exos accelerate wound closure in murine models and improve cardiac repair following myocardial infarction (37, 45–48). Furthermore, Zhang et al. reported that human umbilical cord blood MSC-derived exosomes (UCB-MSC-Exos) promote regenerative wound healing by inhibiting TGF-β receptor signaling (49), while Hao et al. highlighted the significant potential of stem cell exosomes in orofacial wound healing (50). Despite these promising therapeutic achievements, challenges related to isolation, purification, optimization, standardization, and quality control continue to hinder their broad clinical translation (51).

Given the therapeutic potential of stem cell-derived exosomes in cutaneous wound repair and the relatively well-established research foundation in this field, this review briefly outlines the mechanisms of skin wound healing as well as the biogenesis, secretion, and uptake of exosomes, with an emphasis on the molecular mechanisms and latest research advances of adipose-derived stem cell exosomes (ADSC-Exos), bone marrow mesenchymal stem cell exosomes (BMSC-Exos), human umbilical cord mesenchymal stem cell exosomes (hUC-Exos), antler stem cell exosomes (AnSC-Exos), human urine-derived stem cell exosomes (USC-Exos), and induced pluripotent stem cell exosomes (iPSC-Exos) in modulating inflammation, promoting proliferation and migration, stimulating angiogenesis, and inhibiting scar formation. Meanwhile, to comprehensively evaluate the application prospects of exosomes in wound repair, this review also discusses plant-derived exosome-like nanovesicles (PELNs) as supplements and alternatives to stem cell-derived exosomes, systematically examining the latest advances in their therapeutic potential, applications, benefits, and current limitations in cutaneous wound healing.

## 2 Wound healing process

Cutaneous wound healing is a highly coordinated and dynamic biological process aimed at rapidly restoring the skin barrier, preventing infection, and re-establishing tissue integrity. It is classically described as comprising four overlapping yet distinct phases: hemostasis, inflammation, proliferation, and remodeling (15, 52, 53).

**Figure 1:**
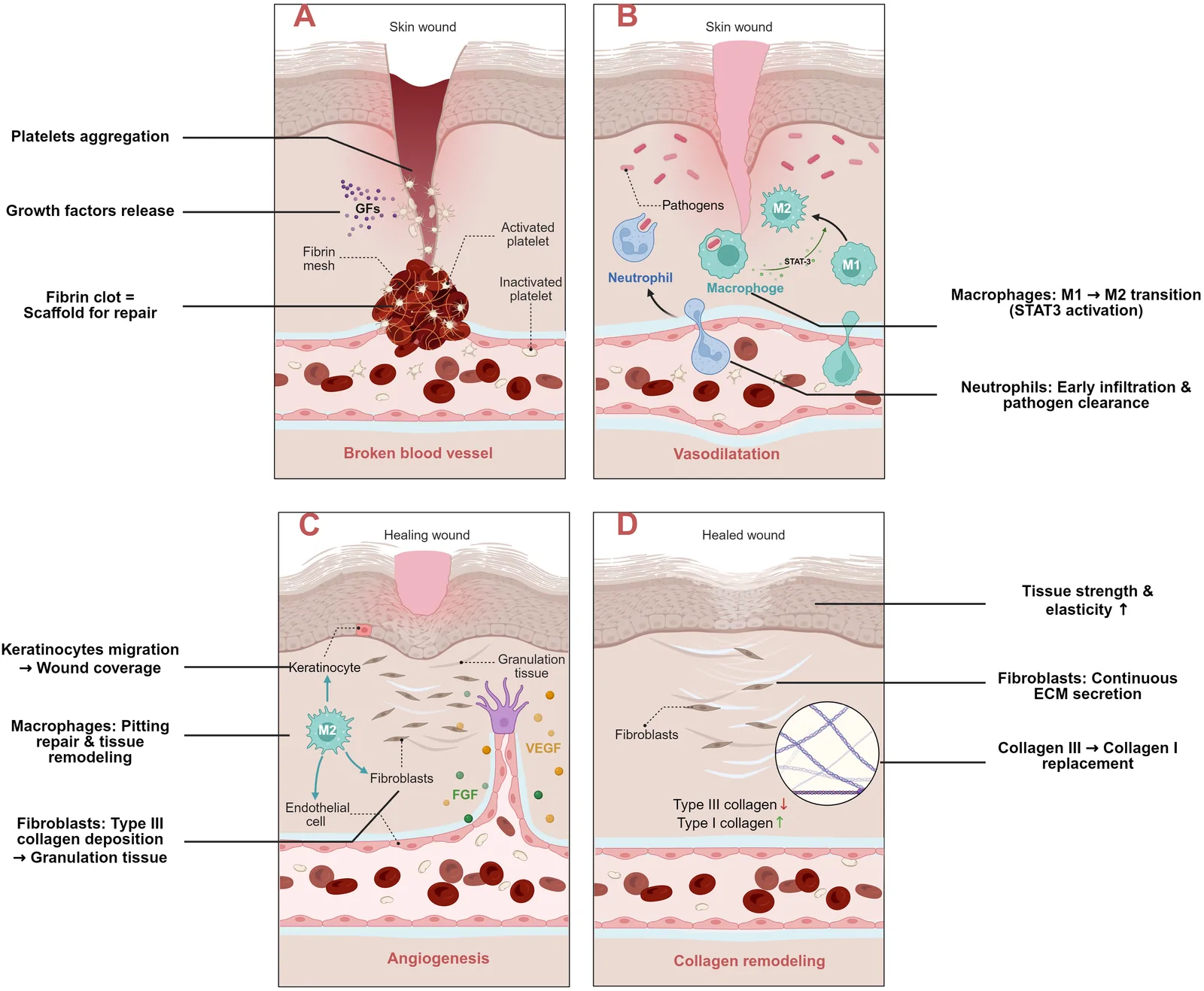
Schematic diagram of the skin wound healing process. **(A)** Hemostasis phase: Platelet aggregation, growth factor release, and fibrin clot formation provide a scaffold for subsequent repair. **(B)** Inflammatory phase: Early infiltration of neutrophils clears pathogens, while macrophages undergo a phenotypic switch from M1 to M2, a process regulated by the STAT3 signaling pathway. **(C)** Proliferation phase: Keratinocytes migrate to re-epithelialize the wound. M2 macrophages participate in repair and tissue remodeling. Fibroblasts secrete type III collagen, forming granulation tissue, with concomitant angiogenesis. **(D)** Remodeling phase: Collagen undergoes remodeling, characterized by the gradual replacement of type III collagen with the more robust type I. Fibroblasts continue to secrete ECM, ultimately enhancing tissue tensile strength and elasticity. The figure was created via BioRender (https://BioRender.com).

The hemostasis phase is triggered immediately upon injury. Damage to the skin leads to vascular rupture, endothelial injury, and exposure of the basement membrane, resulting in hemorrhage. Circulating platelets rapidly adhere to the exposed ECM (e.g., collagen), become activated, and aggregate (54, 55), forming an initial thrombus to seal the vascular breach (**Figure 1A**). During this process, activated platelets undergo degranulation, releasing a multitude of growth factors such as platelet-derived growth factor (PDGF) and TGF-β. Simultaneously, the coagulation cascade is activated, ultimately converting fibrinogen into fibrin, which polymerizes to form a fibrin clot (56). This clot not only achieves effective hemostasis but also acts as a provisional ECM. It provides a scaffold for subsequently infiltrating inflammatory cells and serves as a reservoir for growth factors, thereby initiating the subsequent stages of repair (57, 58).

The inflammatory phase follows closely, typically commencing within hours post-injury and peaking at 24–48 hours. Local tissue damage releases substances such as histamine and bradykinin, inducing vasodilation and increased vascular permeability. This leads to plasma exudation and edema. Neutrophils are the first immune cells recruited to the site, where they phagocytose pathogens and cellular debris. They also secrete various factors, including Signal Transducer and Activator of Transcription 3 (STAT3) (59, 60). Subsequently, monocytes infiltrate the wound and differentiate into macrophages, which play a central regulatory role. These cells often initially exhibit a pro-inflammatory M1 phenotype, characterized by cytotoxic and microbicidal activity and the expression of cytokines such as TNF-α, IL-1β, and IL-6 (61). Later, influenced by microenvironmental signals (e.g., IL-4, IL-13), they typically polarize toward an anti-inflammatory, pro-reparative M2 phenotype (**Figure 1B**). By secreting factors like TGF-β, VEGF, and IL-10, M2 macrophages help resolve inflammation and create a microenvironment conducive to the subsequent proliferation phase, thereby promoting tissue repair (62, 63). The timely and appropriate resolution of inflammation is critical for normal healing progression.

Approximately three days post-injury, the repair process enters the proliferative phase, which is characterized by robust cellular proliferation, migration, and abundant ECM deposition (**Figure 1C**). Angiogenesis proceeds actively, driven by factors including VEGF and Fibroblast Growth Factor (FGF). The resulting nascent capillary network delivers essential oxygen and nutrients to the repairing tissue (64, 65). Fibroblasts, recruited by mediators such as PDGF, migrate into the wound bed, proliferate, and synthesize a provisional ECM rich in type III collagen. This forms the characteristic bright red granulation tissue that fills the wound defect (66). A subset of fibroblasts differentiates into myofibroblasts, which express α-Smooth Muscle Actin (α-SMA) and generate contractile forces responsible for wound contraction (67). Concurrently, keratinocytes at the wound edges become activated, undergo phenotypic changes reminiscent of an epithelial-to-mesenchymal transition (EMT), and proliferate and migrate to cover the newly formed granulation tissue. This process re-establishes a continuous epithelial barrier, crucial for preventing fluid loss and microbial invasion (64).

Finally, wound repair enters the prolonged and critical remodeling phase, which can last from several months to years. The core of this stage is the modification and maturation of the ECM. Fibroblasts continue collagen synthesis, but a new equilibrium between synthesis and degradation is established (68, 69). Collagen metabolism is precisely regulated by matrix metalloproteinases (MMPs) and their tissue inhibitors (TIMPs), which are secreted by fibroblasts and macrophages. As MMPs actively remodel the ECM, overall matrix synthesis gradually slows (70). Consequently, the initial, disorganized type III collagen is progressively degraded and replaced by stronger, highly organized type I collagen fibrils (**Figure 1D****) (**70, 71). Through this process, granulation tissue is progressively converted into scar tissue, and its tensile strength slowly recovers, ultimately reaching a maximum of 80% of that of normal skin. After ECM modification, TIMPs begin to inhibit MMPs, preventing further ECM degradation. However, if inflammation persists or collagen metabolism becomes imbalanced during this process, impaired healing and chronic ulcer formation may result; conversely, if collagen synthesis is excessive while degradation is insufficient, hypertrophic scars or keloids may form (72).

## 3 Exosome

**Figure 2:**
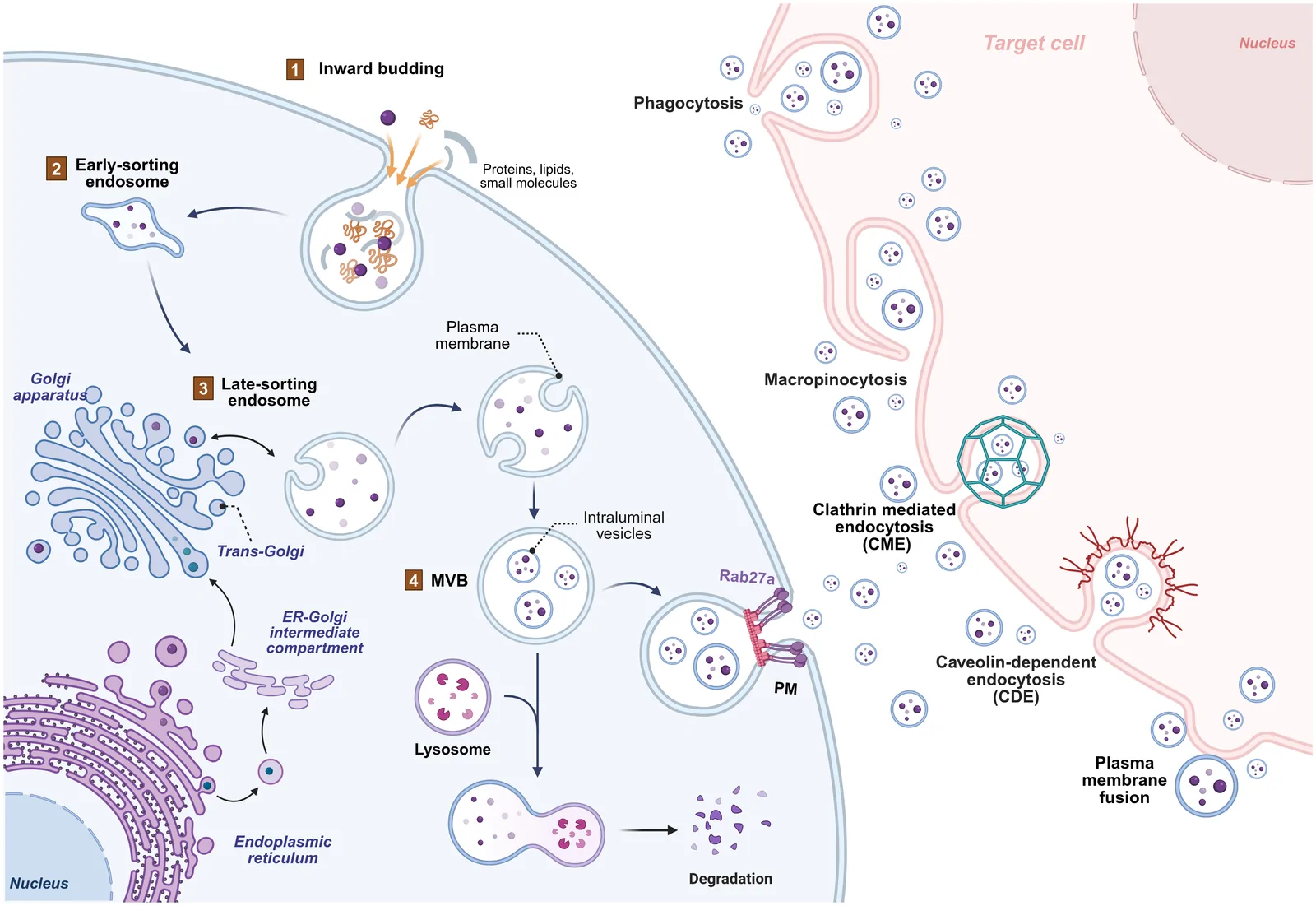
Schematic illustration of exosome biogenesis, secretion, and uptake. Left panel (Donor cell): Early sorting endosomes form via inward budding, which further mature into late sorting endosomes and subsequently give rise to MVBs. MVBs can either fuse with lysosomes for degradation or, under the regulation of Rab27a and other factors, fuse with the plasma membrane to release intraluminal vesicles (exosomes) into the extracellular space. Right panel (Recipient cell): The released vesicles are internalized by the recipient cell through various mechanisms, including phagocytosis, macropinocytosis, clathrin-mediated endocytosis, caveolin-dependent endocytosis, or direct plasma membrane fusion. The figure was created via BioRender (https://BioRender.com).

Exosomes are a class of nanoscale extracellular vesicles secreted by living cells, ranging from 50 to 150 nm in diameter. They are enclosed by a lipid bilayer membrane and contain bioactive molecules such as proteins, nucleic acids (mRNA, miRNA, lncRNA, etc.), and lipids (18, 19). The biogenesis of exosomes begins with the invagination of the plasma membrane to form early sorting endosomes, followed by a second invagination to generate multivesicular bodies (MVBs) containing intraluminal vesicles (ILVs). MVBs face two fates: fusion with lysosomes for degradation (28, 73–75), or fusion with the plasma membrane to release ILVs into the extracellular space, thereby forming exosomes (76–78). This precise process is coordinately regulated by both ESCRT-dependent (79)and ESCRT-independent (80–82)pathways, while the tetraspanin family (CD63, CD9, CD81), as classical markers, participates in the sorting and maturation of vesicles (83–85). After release, exosomes enter the extracellular microenvironment via exocytosis, a process regulated by multiple mechanisms including Rab GTPases, SNARE proteins, and the cytoskeleton (86) (87). Upon reaching target cells, exosomes can be internalized through various pathways such as membrane fusion and endocytosis, thereby enabling intercellular communication (88–90). The processes of exosome biogenesis, secretion, and uptake are illustrated in (**Figure 2**).

### 3.1 Unique advantages of exosomes in treating skin wounds

As a natural nano-delivery system, exosomes exhibit multiple unique advantages in the treatment of skin wounds. First, the anatomical location of the skin offers considerable advantages for the application of exosomes. Unlike other tissues, the skin is located at the surface of the human body, and the resulting wounds can be directly observed and palpated; therefore, exosomes can be readily administered locally to the skin through topical application, spraying, injection, or incorporation into wound dressings (91), without the need for invasive procedures such as endoscopy. Moreover, the healing process of cutaneous wounds is visible to the naked eye, enabling clinicians to intuitively evaluate the therapeutic efficacy of exosomes based on indicators such as wound color, the amount of exudate, granulation tissue growth, contraction rate, and the degree of epithelialization (92). Second, the nanoscale size of exosomes endows them with excellent deep tissue penetration, enabling them to cross natural barriers such as the stratum corneum and basement membrane of the skin to reach the wound bed and exert biological effects (93). Furthermore, as endogenous cellular products, exosomes possess outstanding biocompatibility and low immunogenicity, effectively avoiding the potential cytotoxicity and immune rejection associated with synthetic nanoparticles (94, 95). As a natural “cell-free” therapeutic tool, exosomes carry bioactive molecules from their parent stem cells, mimicking the paracrine effects of stem cells while completely circumventing the tumorigenic risks and ethical concerns associated with live cell transplantation (33).

In terms of targeted delivery, molecules such as integrins and tetraspanins enriched on the exosome surface can specifically recognize ECM components in the injured area, achieving active targeting and accumulation in the wound microenvironment, and crossing the vascular endothelial barrier to precisely deliver cargo to deep damaged regions (96). Notably, exosomes possess multi-target synergistic regulatory capabilities, simultaneously modulating multiple healing processes including inflammatory responses, angiogenesis, cell proliferation and migration, and collagen remodeling (97). Furthermore, exosomes can maintain their bioactivity for long periods at −80 °C, and lyophilized exosomes still retain their functional components, offering significant advantages in storage and transportation for clinical translation (98). Owing to these advantages, exosomes have emerged as a field of great potential in both animal and clinical studies on cutaneous wound healing.

### 3.2 Key bioactive components determining the therapeutic efficacy of exosomes

The therapeutic efficacy of exosomes does not stem from a single component but rather from the synergistic action of multiple bioactive molecules within them. Systematically elucidating these key components is of great significance for optimizing exosome-based therapeutic strategies.

At the protein level, exosomes are rich in growth factors such as VEGF, TGF-β, HGF, and FGF, which respectively regulate angiogenesis, cell proliferation, and ECM synthesis (34). In addition, exosomes carry anti-inflammatory factors such as IL-10 and various chemokines, promoting wound healing by modulating macrophage polarization and reshaping the inflammatory microenvironment (99).

Among the nucleic acid components, miRNAs are the core effector molecules. They function cooperatively and in parallel to coordinate multiple pathways, thereby regulating various stages of healing. As a representative example, miR-126-3p enriched in BMSC-Exos promotes angiogenesis by targeting the angiogenesis inhibitor SPRED1 and activating the Ras/Erk pathway (100). Other functionally important exosomal miRNAs, including miR-21 (101) and miR-146a (102), may also serve as potential therapeutic targets for wound healing.

lncRNAs also constitute important regulatory components, interacting with miRNAs or proteins to construct complex regulatory networks (103). For example, ADSC-Exos-derived lncRNA MALAT1 (Metastasis-associated lung adenocarcinoma transcript-1) promotes fibroblast migration and accelerates the healing of ischemic wounds (104). MSC-derived exosomal lncRNA H19 upregulates gene of phosphate and tension homology deleted on chromosome ten (PTEN) via microRNA-152-3p, thereby promoting wound healing in diabetic foot ulcers (105); and BMSC-derived exosomal lncRNA KLF3-AS1 upregulates VEGFA expression by sponging miR-383, thereby promoting angiogenesis (106).

Furthermore, phosphatidylserine (PS) on the exosomal membrane serves as an important signaling molecule for target cell recognition and internalization, facilitating membrane fusion and accelerating cargo release (107); lipids such as ceramide maintain membrane stability. The multi-level synergistic effects of these four categories of bioactive components, namely proteins, miRNAs, lncRNAs, and lipids, enable exosomes to systematically regulate the entire wound healing process and exhibit a comprehensive therapeutic effect that is difficult to match with single molecules (32).

## 4 The role of stem cell-derived exosomes in wound healing

Numerous studies have established that MSC-Exos can accelerate skin wound healing by mitigating inflammation, promoting neovascularization, and enhancing collagen deposition (97, 108, 109).

**Figure 3:**
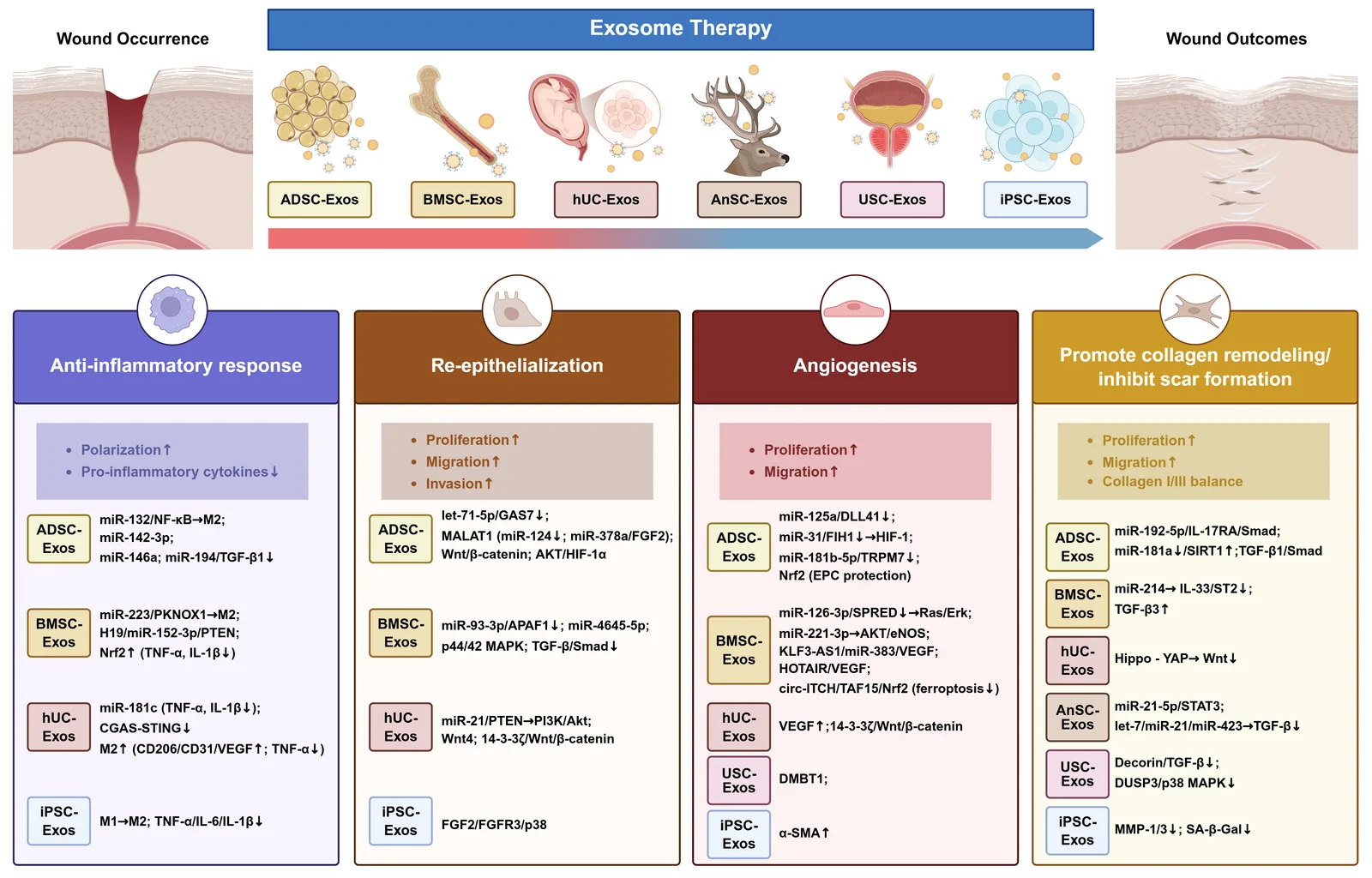
Therapeutic effects and molecular mechanisms of MSC-Exos from various sources in promoting wound healing. The figure was created via BioRender (https://BioRender.com).

Accordingly, MSC-Exos-based therapy has emerged as an important strategy for cutaneous wound repair. This section focuses on the specific roles of ADSC-Exos, BMSC-Exos, and hUC-Exos. Their therapeutic mechanisms include modulating inflammatory responses, promoting cell proliferation and migration, stimulating angiogenesis, and facilitating collagen remodeling while suppressing scar formation. In addition, AnSC-Exos, which have attracted increasing research attention in recent years, together with their emerging potential in wound healing, will also be discussed, as they may offer new perspectives on scarless wound healing. Finally, exosomes derived from stem cells of other tissue sources (USC-Exos and iPSC-Exos) are also summarized (**Figure 3**).

### 4.1 The role of ADSC-exos in wound healing

Adipose-derived stem cells (ADSCs), a type of mesenchymal stem cell, possess the classic multipotency to differentiate into adipogenic, osteogenic, and chondrogenic lineages (110). They offer distinct advantages, including abundant tissue availability, relatively simple isolation, low immunogenicity, and broad therapeutic potential. In recent years, the paracrine function of ADSCs, particularly via their secreted exosomes, has been increasingly recognized as a key mechanism underlying tissue repair and regeneration.

#### 4.1.1 ADSC-exos modulate the inflammatory response

The inflammatory phase is critical for clearing pathogens and debris to establish a microenvironment conducive to regeneration (111). ADSC-Exos exhibit regulatory roles in this phase, though their effects can be context-dependent. While Blazquez et al. reported that ADSC-Exos inhibit pro-inflammatory cytokine secretion by CD4^+^ T cells (112), other studies observed an upregulation of TNF-α and IL-6 (113), highlighting their pleiotropic nature.

Emerging evidence emphasizes the role of non-coding RNAs (ncRNAs) encapsulated in ADSC-Exos. For instance, ADSC-Exos can deliver miR-194 to suppress TGF-β1 in hypertrophic scars, thereby inhibiting inflammatory factor release from myofibroblasts (114). In diabetic wounds, ADSC-Exos overexpressing miR-132 promoted M2 macrophage polarization via the NF-κB pathway (115). Additional mechanisms include anti-inflammatory effects mediated through the upregulation of ROCK1/PTEN and downregulation of miR-124-3p (116), as well as the suppression of pro-inflammatory factors via miR-142-3p and miR-146a (117).

#### 4.1.2 ADSC-exos promote cell proliferation and migration

The proliferative phase relies on the rapid expansion and movement of keratinocytes and fibroblasts to re-epithelialize and contract the wound. ADSC-Exos significantly enhance the viability, migration, and synthetic activity of these cells.

Studies show that ADSC-Exos, especially when combined with hyaluronic acid (HA), synergistically promote wound closure and tissue regeneration *in vitro* and *in vivo (*118). Hu et al. demonstrated that ADSC-Exos enhance fibroblast migration, proliferation, collagen synthesis, and upregulate genes associated with proliferation (N-cadherin, Cyclin-1, PCNA) and collagen I/III expression (32). Notably, they exert a biphasic regulatory effect on collagen, accelerating early repair while preventing excessive scar formation later.

Mechanistically, ADSC-Exos activate the PI3K/AKT (119) and AKT/HIF-1α (120) pathways to promote keratinocyte migration and proliferation. They also improve endothelial cell function by modulating the SIRT3/SOD2 pathway to reduce oxidative stress and inflammation (121), a process potentially involving the eHSP90/LRP1/AKT axis (122). Moreover, ADSC-Exos deliver microRNA let-7i-5p to target and inhibit GAS7, thereby enhancing keratinocyte viability and migration (123). In diabetic models, activation of the TGF-β/Smad3 pathway by ADSC-Exos promotes both fibroblast function and keratinocyte re-epithelialization (124).

The Wnt/β-catenin pathway is another key target (125). ADSC-Exos activate this pathway to promote proliferation and migration while inhibiting apoptosis in oxidatively damaged HaCaT cells, suggesting utility in age-related chronic wounds (126).

MALAT1 is a widely studied lncRNA, known for its ability to reduce pro-inflammatory cytokine levels (127). The lncRNA MALAT1 within ADSC-Exos plays a multifaceted role. It promotes human dermal fibroblasts (HDFs) migration and accelerates ischemic wound healing (103), activates the Wnt/β-catenin pathway while inhibiting miR-124 to alleviate apoptosis (128), and modulates the miR-378a/FGF2 axis to enhance repair (129).

#### 4.1.3 ADSC-exos promote angiogenesis

Adequate neovascularization is essential for delivering oxygen and nutrients during healing (58). The transcription factor nuclear factor erythroid 2-related factor 2 (Nrf2) confers protection against oxidative stress, and its deficiency exacerbates endothelial dysfunction in diabetic foot ulcers (DFUs) (130). High glucose conditions impair DFU healing by inducing endothelial progenitor cell (EPC) senescence, enhancing oxidative stress, and promoting inflammation. Conversely, ADSC-Exos overexpressing Nrf2 can protect EPCs from high glucose-induced damage and promote angiogenesis by delivering antioxidant signals (131). In a study on flap ischemia-reperfusion injury, ADSC-Exos preconditioned with H_2_O_2_ demonstrated a superior ability to promote blood vessel formation and enhance flap survival compared to conventional ADSC-Exos (132).

At the molecular level, ADSC-Exos deliver miR-125a to endothelial cells, where it targets and inhibits the angiogenesis suppressor DLL4, facilitating tip cell formation (133). Similarly, miR-181b-5p transferred via ADSC-Exos promotes angiogenesis in brain endothelial cells after injury by targeting TRPM7 (134). Furthermore, exosomal miR-31 inhibits FIH1 in endothelial cells, thereby activating the HIF-1 pathway to stimulate blood vessel growth (135).

#### 4.1.4 ADSC-exos promote collagen remodeling or inhibit scar formation

Ideal wound healing should achieve regeneration with minimal or no scarring. However, in most mammals, the healing process is often accompanied by fibrosis and scar formation, which can compromise both tissue function and aesthetics (58). Scar formation is characterized by the activation of various pro-fibrotic factors within the scar tissue, ultimately leading to the excessive deposition and altered architecture of collagen types I and III (136).

ADSC-Exos carry various anti-fibrotic components that modulate fibroblast activity. They can attenuate scarring through the miR-192-5p/IL-17RA/Smad axis (137) and the TGF-β1/Smad signaling pathway (138–140). Wang et al. discovered that ADSC-Exos inhibit the differentiation of fibroblasts into myofibroblasts (141), which is conducive to ECM remodeling and contributes to reducing scarring. Furthermore, Chen et al. reported that ADSC-Exos attenuate collagen deposition and α-SMA production in fibroblasts by downregulating miR-181a and upregulating SIRT1 expression (142).

### 4.2 The role of BMSC-exos in wound healing

Bone marrow mesenchymal stem cells (BMSCs) represent the most extensively utilized and researched type of mesenchymal stem cells (MSCs). Initially discovered in the 1960s by Ernest A. McCulloch and James E. Till, BMSCs served as a foundational cell source for clonal studies (143). Throughout the 1970s and 1980s, the potential of BMSCs was significantly explored. In subsequent decades, extensive and systematic research has been conducted to unlock the therapeutic potential of BMSCs for various diseases.

#### 4.2.1 BMSC-exos modulate the inflammatory response

BMSC-Exos preconditioned with melatonin can alleviate the inflammatory response and significantly improve healing outcomes by promoting the polarization of macrophages from the pro-inflammatory M1 phenotype towards the anti-inflammatory M2 phenotype. This effect is mediated through the activation of PTEN and subsequent inhibition of AKT phosphorylation (144). Similarly, miR-223 within BMSC-Exos can target PKNOX1, an inhibitor of M2 polarization, to facilitate the macrophage phenotype switch, thereby promoting anti-inflammation and tissue repair (145).

Another study demonstrated that BMSC-Exos also promote fibroblast proliferation and migration while inhibiting apoptosis and the inflammatory response, ultimately accelerating diabetic foot ulcer healing. This occurs through the upregulation of PTEN expression and inhibition of the PI3K/Akt signaling pathway. In this process, the lncRNA H19 acts as a molecular sponge for miR-152-3p, relieving its inhibitory effect on PTEN (104). Furthermore, BMSC-Exos suppress the inflammatory response in diabetic wounds by upregulating Nrf2 and subsequently downregulating TNF-α and IL-1β. The combination with the Nrf2 activator tBHQ significantly enhanced this effect, suggesting a potential effective strategy for treating diabetic chronic wounds (146).

#### 4.2.2 BMSC-exos promote cell proliferation and migration

BMSC-Exos demonstrate the capacity to promote the proliferation of keratinocytes and endothelial cells, enhancing overall cell viability. Similar to ADSC-Exos, they also stimulate the migration of both fibroblasts and keratinocytes (147). The promotive effects of BMSC-Exos are mediated through specific molecular pathways. For instance, they accelerate corneal wound healing significantly by activating the p44/42 MAPK signaling pathway, thereby enhancing the proliferation and migration of corneal epithelial cells (148). Furthermore, human BMSC-Exos can induce the proliferation of HaCaT cells and HDFs by inhibiting the TGF-β/Smad pathway, ultimately enhancing the wound healing process (149).

Hypoxic preconditioning augments the therapeutic potential of BMSC-Exos. Exosomes derived from hypoxia-preconditioned BMSCs significantly accelerate diabetic wound closure and enhance keratinocyte proliferation compared to those from normoxic conditions, with miR-4645-5p implicated as a functional mediator (150). BMSC-Exos also exert their effects via specific miRNA cargo. For instance, miR-146a-enriched BMSC-Exos enhance re-epithelialization, angiogenesis, and M2 macrophage polarization in diabetic wounds (151). Similarly, Shen et al. reported that BMSC-Exos, enriched with miR-93-3p, restore the proliferation and migration capacity of damaged HaCaT cells and inhibit their apoptosis. This effect is achieved by targeting and downregulating the pro-apoptotic factor APAF1, finally promoting skin wound healing (152).

#### 4.2.3 BMSC-exos promote angiogenesis

Endothelial nitric oxide synthase (eNOS) plays a pivotal role in wound healing, with its concentration positively correlating with capillary formation during the process (153). BMSC-Exos derived from cells pretreated with atorvastatin (ATV-Exos) promote re-epithelialization and ECM remodeling, thereby accelerating wound healing in a diabetic rat model (154). *In vitro*, ATV-Exos stimulate angiogenesis by upregulating miR-221-3p, which subsequently activates the AKT/eNOS pathway (154). Similarly, exosomes from pioglitazone-pretreated BMSCs (PGZ-Exos) inhibit PTEN and activate the PI3K/AKT/eNOS axis, enhancing endothelial cell function and angiogenesis (155).

Pretreatment with interferon-gamma (IFN-γ) significantly enhances the therapeutic efficacy of BMSC-Exos. These exosomes, enriched with miR-126-3p, promote angiogenesis by targeting and suppressing the angiogenesis inhibitor SPRED1, thereby activating the Ras/Erk pathway (101). Consistently, BMSC-Exos overexpressing miR-126 enhance endothelial cell proliferation, migration, and angiogenic capacity *in vivo* by targeting PIK3R2 and activating the PI3K/Akt signaling pathway, significantly accelerating wound healing (156).

Other molecular players include the lncRNA KLF3-AS1, which acts as a sponge for miR-383 to upregulate VEGF and promote angiogenesis (105), and BMSC-Exos loaded with a miR-155 inhibitor, which upregulate FGF-7 and VEGF to enhance vascularization (157). Circular RNA circ-ITCH, delivered by BMSC-Exos, recruits TAF15 to activate the Nrf2 pathway, restoring endothelial cell function and inhibiting ferroptosis (158). Similarly, exosomal HOTAIR upregulates VEGF and promotes angiogenesis in diabetic models (159).

The AKT/ERK1/2/STAT3 pathway is also activated by BMSC-Exos, leading to insulin-like growth factor-1 (IGF1) upregulation and enhanced angiogenesis. The protein Insig1 promotes VEGF expression and angiogenesis by enhancing BMSC-Exos secretion, with miR-132–3p serving as a key mediator in this process (160).

#### 4.2.4 BMSC-exos promote collagen remodeling or inhibit scar formation

Systemic sclerosis is a disease characterized by excessive skin fibrosis, massive collagen deposition, and abnormal proliferation and migration of fibroblasts. Xie et al. demonstrated that BMSC-Exos deliver miR-214 to block the IL-33/ST2 pathway, thereby alleviating skin sclerosis and fibrosis (161). Jiang et al. found that BMSC-Exos increase TGF-β3 expression, which may promote scarless healing of skin wounds (149).

### 4.3 The role of hUC-exos in wound healing

Human umbilical cord mesenchymal stem cells (hUC-MSCs) offer distinct advantages over those derived from bone marrow or adipose tissue, including low immunogenicity, non-invasive harvest, ease of expansion, and fewer ethical concerns (162). Consequently, hUC-Exos have attracted growing research interest in regenerative medicine.

In a diabetic mouse model, hUC-Exos delivered via a thermosensitive hydrogel suppressed inflammation by inhibiting the cGAS-STING signaling pathway while concurrently upregulating VEGF expression to promote angiogenesis (163). Furthermore, a chitosan-based liquid bandage loaded with hUC-Exos directly stimulated the proliferation and migration of human umbilical vein endothelial cells (HUVECs), accelerating angiogenesis without inducing systemic toxicity. It also modulated the local inflammatory environment, collectively promoting wound healing (164). Li et al. demonstrated that hUC-Exos enriched with miR-181c inhibited the secretion of pro-inflammatory cytokines (TNF-α and IL-1β), facilitating healing in a rat model of severe burns (165). Similarly, Teng et al. reported that hUC-Exos reduced oxidative stress and promoted an M2 macrophage phenotype (upregulated CD206, CD31and VEGF; downregulated TNF-α), accelerating diabetic wound repair (166).

A study in a diabetic mouse model demonstrated that hUC-Exos significantly accelerated wound healing, outperforming conventional treatments by ameliorating oxidative stress and promoting angiogenesis (167). Hypoxic preconditioning further augments their efficacy; exosomes from hypoxia-treated hUC-MSCs promoted fibroblast activity *in vitro* and enhanced re-epithelialization and collagen synthesis *in vivo (*168). Beyond epithelial and mesenchymal cells, Zhu et al. reported that hUC-Exos can stimulate fibroblasts to secrete Nerve Growth Factor (NGF), suggesting a potential role in promoting skin nerve regeneration (169).

The therapeutic effects of hUC-Exos are mediated through precise modulation of critical signaling pathways. They deliver miR-21 to inhibit PTEN and activate the PI3K/Akt pathway, thereby accelerating corneal epithelial cell proliferation and migration (170). Activation of the Wnt/β-catenin pathway is crucial for their pro-healing effects, as knockdown of Wnt4 suppressed skin cell proliferation and migration (171). Furthermore, hUC-Exos promote HaCaT cell proliferation and migration by inhibiting the nuclear translocation of Apoptosis-Inducing Factor (AIF) and upregulating PARP-1 expression (172).

Notably, a temporally regulated dual-pathway mechanism was identified in a rat burn model. In early stages, hUC-Exos deliver 14-3-3ζ to activate the Wnt/β-catenin pathway, promoting cell proliferation, re-epithelialization, and angiogenesis. In later stages, they activate the Hippo-YAP pathway to subsequently inhibit Wnt signaling, thereby preventing excessive proliferation and collagen deposition to minimize scar formation (173).

### 4.4 The role of AnSC-exos in wound healing

Among mammals, deer antlers are currently the only known organs capable of complete regeneration (174–176). In spring, hard antlers are cast from the pedicle (the permanent bony base). Subsequently, antler bone and cartilage regenerate from the pedicle periosteum located at the pedicle stump. During summer, the antlers undergo rapid growth, with studies indicating growth rates reaching up to 2 cm/day (177). By late summer/early autumn, growth slows. In deep autumn, the antlers become fully calcified and the velvet (skin covering) is shed. Throughout winter, the fully calcified antlers remain firmly attached to the pedicles. Finally, in the following spring, the calcified antlers are shed completely, initiating a new cycle of regeneration (178–180). This scarless regeneration and healing relies on PRRX1^+^ mesenchymal cells (PMCs) within the pedicle periosteum or Antler Stem Cells (AnSCs) present in the early antler blastema (181, 182), These stem cells exhibit sustained self-renewal and can differentiate into multiple lineages to form the complex organ (181). Compared to other mammalian stem cell types, AnSCs are more accessible and demonstrate superior proliferative and regenerative capacities (183, 184).

Currently, studies have demonstrated that AnSC-Exos promote wound healing in murine wound models through the regeneration of skin appendages, upregulation of type III collagen expression, downregulation of type I collagen expression, and inhibition of fibroblast-to-myofibroblast transition (FMT), potentially mediated by let-7, miR-21, and miR-423-mediated suppression of TGF-β signaling to inhibit FMT (185). Meng et al. found that AnSC-Exos effectively promote skin wound healing in mice, and the miR-21-5p/STAT3 axis may play a key role in this process (178).

### 4.5 Role of human USC-exos in wound healing

Human urine-derived stem cells (USCs) are stem cells isolated from human urine; the isolation procedure is simple and cost-effective and, more importantly, requires no invasive procedures for cell acquisition. USCs exhibit remarkable proliferative capacity and multilineage differentiation potential, and have emerged in recent years as a novel stem cell source in the field of tissue regeneration. As early as 2014, it was reported that human urine-derived stem cells, in combination with polycaprolactone/gelatin nanofibrous membranes, significantly promoted the healing of full-thickness skin wounds in rabbits (186). A subsequent study constructed a small intestinal submucosa composite graft loaded with USCs and subjected it to hypoxic preconditioning, which enhanced the expression of wound angiogenesis-related genes (VEGF and Ang-2) and epithelialization-related genes basic fibroblast growth factor (bFGF) and epidermal growth factor (EGF), while also promoting the secretion of growth factors (VEGF, EGF, and bFGF) (187).

Regarding human USC-Exos, Chen et al. found that USC-Exos enhanced angiogenesis and promoted diabetic wound repair by delivering the pro-angiogenic protein DMBT1 (188). USC-derived small extracellular vesicles were shown to modulate the TGF-β and p38 MAPK signaling pathways by transferring decorin and DUSP3 proteins, respectively, thereby inhibiting fibrosis and promoting scarless healing of vaginal defects in rabbits.

### 4.6 The role of iPSC-exos in wound healing

Induced pluripotent stem cells (iPSCs) are pluripotent stem cells typically generated from somatic cells through a defined panel of transcription factors (189) and possess exceptional differentiation capacity. iPSC-Exos combine the advantages of MSCs and iPSCs, exhibiting both embryonic stem cell-like biological activity and low immunogenicity (190), making them a promising source for cell therapy (191). Kobayashi et al. first demonstrated that iPSC-Exos could promote wound healing in diabetic mice, significantly increasing the proliferation and migration of HDFs (190). Similarly, Zhang et al. demonstrated that iPSC-Exos promote the proliferation and migration of HaCaT cells, an effect that may depend on the FGF2/FGFR3/p38 pathway (192). In the field of skin aging, studies have likewise found that iPSC-Exos stimulate the proliferation and migration of HDFs and significantly reduce the expression of SA-β-Gal and MMP-1/3, demonstrating the considerable potential of iPSC-Exos for the treatment of skin aging (193). Long et al. demonstrated that iPSC-Exos may promote wound healing through multiple mechanisms, including enhanced re-epithelialization and angiogenesis (increased α-SMA expression), reduced levels of inflammatory cytokines (TNF-α, IL-6, and IL-1β), and promotion of macrophage polarization from the M1 to the M2 phenotype (194). Collectively, these studies suggest that iPSC-Exos hold great promise as a prospective option for skin rejuvenation and regeneration.

## 5 PELNs: a complementary and expanding perspective

Exosomes are not exclusive to animals; nanovesicles with exosome-like characteristics can also be isolated from plants and are commonly referred to as PELNs (195, 196). PELNs are exosome-like nanoscale vesicles with diameters ranging from 50 to 500 nm, enclosed by a lipid bilayer membrane and containing proteins (197, 198), nucleic acids (199, 200), lipids (201), and other small molecules. These constituents play crucial roles in cellular signal transduction and the regulation of gene expression.

Before discussing specific plant sources, it is necessary to clarify the conceptual boundaries between PELNs and mammalian-derived exosomes (MDEs), as the two exhibit both similarities and differences at certain levels. First, at the stage of biogenesis, current mainstream research holds that the MVB pathway is the principal route of PELN formation, which is highly similar to that of MDEs (202). Second, with regard to biochemical composition, although the membrane of PELNs, like that of MDEs, is composed of a phospholipid bilayer, PELNs are enriched in plant-specific lipids, such as high concentrations of phosphatidic acid, phosphatidylcholine, digalactosyldiacylglycerol, and monogalactosyldiacylglycerol (203), along with lower protein concentrations. Third, in terms of surface marker proteins, PELNs generally lack the tetraspanin markers that define MDEs, such as CD63, CD9, and CD81. Fourth, regarding extraction and purification, although PELNs offer advantages such as high yield, research on their extraction and purification currently lags behind that of MDEs, and no standardized isolation procedure for PELNs has been established. Finally, because PELNs are typically derived from edible or medicinal plants, they are often assumed to be intrinsically safe; although studies have shown that PELNs are orally administrable and carry no risk of zoonotic transmission (204), systematic toxicological, immunogenicity, and biodistribution data remain limited.

Therefore, consistent with current nomenclature in the field, these vesicles are referred to as PELNs or plant-derived exosomes throughout this review. Against this background, this review positions PELNs as a complementary and expanding perspective in vesicle-based wound therapy. To date, PELNs have been isolated from various parts of plants and, as an emerging class of natural nanotherapeutics, have become a hot research topic (201, 205–208).

### 5.1 Aloe-derived exosome-like nanovesicles

Aloe is a well-established plant with recognized cosmetic and therapeutic value. Its antioxidant properties contribute significantly to its potential in promoting skin wound healing. Studies indicate that Aloe-derived exosome-like nanovesicles (A-ELNs) accelerate wound closure by enhancing the migration of keratinocytes and fibroblasts, a process in which the transcription factor Nrf2 plays a key regulatory role (209). Regarding their anti-inflammatory effects, A-ELNs reduce the secretion of inflammatory cytokines by inhibiting the NF-κB pathway, thereby mitigating excessive inflammation in burn wounds. They also suppress the transformation of fibroblasts into pro-fibrotic myofibroblasts (210). Similarly, Kim et al. reported that A-ELNs significantly inhibit the mRNA expression of the pro-inflammatory cytokines IL-6 and IL-1β. Furthermore, they promote the formation of capillary-like structures by HUVECs, enhancing angiogenesis and thereby improving blood supply to the wound site (211). Beyond single-plant extracts, combined formulations show synergistic potential. Miya et al. developed a mixture of exosome-like nanovesicles from Aloe, Zingiber officinale (ginger) juice, and Azadirachta indica (neem) fruit extract, which significantly reduced healing time in a diabetic wound model (212). Follow-up work by the same team demonstrated that this biomaterial suppresses the expression of MMP-2/9 while increasing TGF-β levels, resulting in enhanced collagen deposition and keratinocyte proliferation (213).

### 5.2 Panax ginseng-derived exosome-like nanovesicles

Panax ginseng is a globally utilized natural product and a valued herb in traditional medicine, with Asian ginseng and American ginseng being its two primary varieties (214). The key bioactive constituents of ginseng are dammarane-type saponins, commonly known as ginsenosides, which exhibit a broad spectrum of pharmacological activities (215). Increasingly, their potential in dermatological applications is being explored.

You et al. summarized the multifaceted effects of Korean ginseng, including anti-photoaging, anti-inflammatory, antioxidant, anti-melanogenic, and wound healing activities, and outlined future research directions for ginseng-derived components in skin health (216). At the vesicular level, ginseng-derived exosome-like nanovesicles (G-ELNs) and ginseng-Derived Extracellular Vesicles (G-Evs) have shown significant bioactivity. Guo et al. reported that G-ELNs exert potent anti-tumor effects by upregulating pro-inflammatory cytokines such as TNF-α and IL-12 (217). In the context of skin aging, Cho et al. demonstrated the anti-aging properties of G-Evs (218), and Choi et al. found that G-ELNs confer anti-aging benefits by targeting the MEK1/2-AP-1 pathway, thereby inhibiting skin apoptosis, aging, and inflammation (219). Furthermore, Yang et al. showed that G-ELNs activate the ERK and AKT/mTOR signaling pathways while significantly suppressing inflammatory responses (220). Separately, a study in a diabetic mouse wound model highlighted the potent pro-angiogenic effect of these nanovesicles *in vivo* (221).

Emerging clinical observations support their translational potential. In a case report involving two patients with post-inflammatory hyperpigmentation, microneedling combined with topical ginseng-derived exosomes application led to significant fading of hyperpigmentation and improved skin texture, with no adverse effects reported (222).

In summary, G-ELNs/G-Evs not only promote wound healing but also exhibit anti-aging properties. This dual functionality underscores their considerable potential and applicability in the field of skin regeneration and repair.

### 5.3 Zingiber officinale (ginger)-derived exosome-like nanovesicles

Zingiber officinale (ginger) is both a common culinary ingredient and a traditional medicinal herb, long valued in Chinese culture for its notable anti-inflammatory properties. Its active constituents, such as gingerols and shogaols, have been confirmed *in vitro* and *in vivo* to possess anti-inflammatory, antioxidant, and antiemetic activities. Clinically, ginger is used to manage pregnancy-related nausea, support diabetes care, and mitigate chemotherapy-induced side effects in cancer patients (223).

Ginger-derived exosome-like nanovesicles (Gin-ELNs) have recently emerged as a novel natural platform for disease therapy and as biocompatible nanocarriers for drug delivery, with applications in oncology (224, 225), gut microbiota modulation (226), and anti-colitis therapy (203, 227).

In dermatology, Gin-ELNs show considerable promise for wound healing and tissue regeneration. Ultraviolet A (UVA) radiation (320–400 nm), which accounts for up to 95% of solar UV exposure, is well-established to cause significant skin damage and contribute to various pathological processes (228). In this context, a thermosensitive hydrogel loaded with ginger vesicles has demonstrated efficacy in protecting skin cells from UVA-induced damage through reactive oxygen species (ROS) scavenging (229).

Furthermore, in a rat model following osteosarcoma resection, ginger vesicles co-loaded with doxorubicin (DOX) were incorporated into a hydrogel composed of carboxymethyl chitosan methacryloyl (CMCSMA) and tannic acid (TA). This composite hydrogel significantly shortened hemostasis time, reduced tumor recurrence, and accelerated wound healing compared to conventional sponge dressings (230).

### 5.4 Allium sativum (garlic)-derived exosome-like nanovesicles

Allium sativum (garlic) is a widely used spice, valued for its distinct flavor and aroma, as well as its rich content of bioactive compounds that contribute to disease prevention and human health (231). Both garlic and its derived exosomes exhibit potent anti-inflammatory activity (232) and the ability to remodel gut microbiota (233). In skin repair, garlic-derived exosomes have been shown to promote hair growth in rats by activating the Wnt-1/β-catenin pathway and upregulating VEGF, PDGF, TGF-β1, and Collagen I expression (234). Moreover, vancomycin-loaded garlic exosome-like nanovesicles effectively eradicated Staphylococcus aureus in infected wounds (235). Kalarikkal et al. further reported that garlic- and shallot-derived nanovesicles synergistically exert anti-inflammatory and pro-epidermal differentiation effects through dual modulation of the Nrf2 pathway and the IL-17 axis (236).

### 5.5 Grape-derived exosome-like nanovesicles

Research on other plant-derived vesicles also highlights their dermatological potential. Grape-derived exosome-like nanovesicles (Gra-ELNs) significantly promoted HaCaT cell proliferation, reduced inflammation, and enhanced tube formation in HUVECs (237). Combined *in vitro* and *in vivo* studies indicated that Gra-ELNs markedly reduce UV-induced skin damage and promote skin repair (238). In a randomized, double-blind, split-face trial, Lycium barbarum (goji berry)-derived exosome-like nanovesicles improved skin barrier function, as evidenced by reduced transepidermal water loss and increased stratum corneum thickness (239).

### 5.6 Dendrobium-derived exosome-like nanovesicles

Dendrobium officinale has long been recognized as a precious herbal medicine. Its medicinal value and market demand have attracted extensive research interest (240). Modern pharmacological studies have confirmed that Dendrobium possesses various functions, including anti-tumor, antioxidant, anti-aging, anti-inflammatory, and immunomodulatory activities (240). Tu et al. found that Dendrobium-derived exosome-like nanovesicles inhibit IL-1β and reduce inflammatory cell infiltration (241). Subsequent work from the same group identified the Akt/eNOS signaling pathway as a key mediator of their pro-angiogenic and ECM-remodeling effects, thereby accelerating wound healing (242).

### 5.7 Other PELNs

In addition to the well-studied medicinal plants such as Aloe, Ginseng, Ginger, Garlic, Grape, and Dendrobium, numerous other traditional Chinese herbs have been shown to accelerate skin wound healing. Their mechanisms of action include promoting fibroblast proliferation and migration (243, 244), downregulating the expression of inflammatory cytokines (245–248), inducing macrophage polarization toward the reparative M2 phenotype (249), and stimulating angiogenesis (250).

The Nrf2 signaling pathway appears to be a central mediator in the skin-healing effects of many traditional herbs. For instance, turmeric-derived exosome-like nanovesicles activate the Nrf2 pathway to promote fibroblast proliferation and migration. They also reduce the expression of inflammatory factors such as IL-1β and IL-6 and shift macrophage polarization from the M1 to the M2 phenotype, thereby accelerating diabetic wound healing (251). Similarly, lemon-derived nanoparticle-functionalized hydrogels can remodel macrophage polarization (M1→M2), promote tube formation in HUVECs, and enhance fibroblast activity, effectively improving diabetic wound repair. This process is associated with a significant increase in eNOS expression (252), likely mediated through the inhibition of NF-κB signaling. Coriander-derived exosome-like nanovesicles also activate the Nrf2 pathway and suppress IL-1β and TNF-α expression, significantly shortening wound closure time in a mouse model (253). These vesicles are internalized by HaCaT cells and mouse skin tissue, where they promote cell migration and ultimately accelerate healing (253).

In summary, PELNs constitute a complementary and alternative vesicle source. Their broad availability, low immunogenicity, and low production cost render them an attractive candidate, particularly suitable for topical application and resource-limited settings, while their unique biogenesis and compositional characteristics necessitate the establishment of dedicated standards for isolation, characterization, dosage, and safety evaluation. Before PELNs can be firmly established in the field of skin regenerative medicine, systematic mechanistic and toxicological studies are still required.

## 6 Clinical applications and future perspectives of exosome therapy for skin diseases

Skin wound healing is a complex, tightly regulated physiological process that requires coordinated cellular and molecular interactions. Conventional treatments often show limited efficacy for complex or chronic wounds. In this context, stem cells, their derived exosomes, and plant-derived exosomes have emerged as promising regenerative strategies in recent years. Clinical evidence increasingly supports the safety, feasibility, and effectiveness of exosome-based therapies for wound healing and skin regeneration in humans (**Table 1**).

**Table 1:** Clinical applications of stem cells, stem cell–derived exosomes, and plant-derived exosomes in clinical trials.

Condition	Cell source	Results	References
Skin wounds in healthy volunteers	Activated human platelets	Promoted proliferation and migration of dermal fibroblasts and angiogenesis of human dermal endothelial cells; safe and well tolerated, with no serious adverse events	(254)
DFU	hUC-MSCs	Increased neovessel formation, with complete or progressive healing of foot ulcers and no serious complications	(255)
DFU	Autologous BMSCs and human skin fibroblasts (HSF)	Sustained reduction in wound area, increased dermal vascular density in the wound bed, and greater dermal thickness	(256)
DFU	Umbilical cord Wharton’s jelly–derived mesenchymal stem cells (WJSCs)	Significantly shortened wound-healing time and reduced wound area	(257)
Acute graft-versus-host disease (aGVHD)	BMSCs	Complete or partial remission according to treatment duration	(258)
aGVHD	Ex vivo–cultured adult human mesenchymal stem cells (Prochymal™)	Complete remission in 7 patients and partial remission in 2; complete resolution of gastrointestinal symptoms in 9 patients	(259)
Skin wrinkles and hyperpigmentation	Adipocyte-derived stem cell-containing medium (ADSC-CM)	Skin regeneration via downregulation of MMP-1 and MMP-2 and upregulation of type I collagen	(260)
Skin erythema and hyperpigmentation	Human umbilical cord blood-derived mesenchymal stem cell conditioned media	Improved erythema and overall skin texture, shortened post-laser recovery, and no adverse reactions	(261)
Atrophic acne scars	ADSCs	Significant reductions in scar depression volume, pore volume, and skin roughness	(262)
Skin wrinkles, loss of elasticity, and hyperpigmentation	ADSCs	Improved recovery of skin elasticity, increased stratum corneum hydration, and reduced pigmentary unevenness, with no serious adverse events observed	(263)
Post-inflammatory hyperpigmentation (PIH) following acne	Panax ginseng stem cell	Marked lightening of hyperpigmentation and a more even skin tone, high patient satisfaction, and no adverse reactions	(222)
Photoaged facial skin	ADSCs	Increased type I collagen and glycosaminoglycan content	(264)
Deep second-degree burn of the left thigh	hUC-MSCs	Accelerated re-epithelialization with minimal scarring	(265)
Nonhealing Scalp Wound	Platelets	Complete healing of the frontal wound and a 96% reduction in the overall size of the temporoparietal wound	(267)
Skin graft donor-site wounds	Platelets	100% re-epithelialization with a markedly shortened healing time	(268)
Ischemic nasal flap wound	Rose	Complete skin regeneration within 30 days	(269)
Ischemic Auricular Flap wound	Rose	Complete skin regeneration within 14 days	(270)
Acneiform rash and pruritus	Rose	Near-complete resolution of inflammation within 72 h, with restoration of normal skin appearance	(271)
Skin after radiofrequency microneedling	Rose	Improved skin wrinkles, texture, and pore size	(272)

Initial clinical studies have reported some positive signals. Johnson et al. first reported the safe use of human platelet-derived exosomes for wound healing, with no serious adverse events or clinically significant abnormalities observed over 30 days (254). In patients with DFUs, interventions involving stem cells have shown tangible benefits. For instance, transplantation of hUC-MSCs following angioplasty in 53 DFU patients led to marked neovascularization and complete or partial ulcer healing within three months (255). Another DFU study combined an autologous dermal fibroblast-seeded collagen scaffold with autologous bone marrow-derived MSCs, resulting in sustained wound area reduction, increased dermal vascular density, and thickness after 29 days (256). Similarly, application of a decellularized amniotic membrane covered with Wharton’s jelly MSCs significantly reduced chronic diabetic wound size within 9 days (257). For steroid-refractory acute graft-versus-host disease (aGVHD), a severe post-transplant complication, MSC infusion has provided a new therapeutic avenue (258, 259).

Beyond chronic wounds, exosome-based approaches show potential in managing other skin conditions. A study on 24 patients with skin aging treated with ablative fractional laser (AFL) showed that adipose-derived stem cell conditioned medium (ADSC-CM) combined with niacinamide exerts anti-aging effects by reducing the expression of MMP-1 and MMP-2 and promoting the increased expression of type 1 collagen (260). Another study demonstrated that a topical formulation containing serum and cream prepared from allogeneic hUC-MSCs accelerated the wound healing process and mitigated post-treatment erythema following AFL therapy. This finding confirms the potential of this treatment in supporting tissue repair after laser procedures (261).

Kwon et al. conducted a 12-week split-face trial using human adipose stem cell-derived exosome gel combined with fractional CO_2_ laser for atrophic acne scars, reporting satisfactory efficacy compared to the control gel (262). In another study by the same group, a solution containing adipose stem cell exosomes applied with microneedling improved skin elasticity, hydration, and pigmentation homogeneity in patients with facial aging over 12 weeks, with a favorable safety profile (263).

A separate clinical trial demonstrated the efficacy and safety of ADSC-derived exosomes ADSC-Exos in photoaged facial skin, showing that ADSC-EXO treatment increased the levels of type I collagen and glycosaminoglycans (264). Hung et al. reported the therapeutic effect of hUC-Exos in the treatment of cutaneous burns (265).

Purified exosome product (PEP) is an exosome product isolated from platelets (266). In a case report of a non-healing scalp wound following chemoradiotherapy, the patient received four treatments of collagen–PEP and four treatments of fibrin–PEP over a 7-month period, resulting in complete healing of the frontal wound and a 96% reduction in the overall size of the temporoparietal wound (267). Similarly, in skin graft wounds, PEP treatment achieved complete wound healing(100% re-epithelialization), with a markedly shortened healing time (268). These case reports provide compelling evidence for the therapeutic potential of PEP in wound repair.

In addition, plant-derived exosomes have also demonstrated therapeutic value in skin diseases. A case report showed that microneedling combined with topical application of ginseng-derived exosomes significantly improved post-inflammatory hyperpigmentation (222). Carvajal’s group reported a patient with an ischemic nasal flap wound who achieved complete skin regeneration of the nasal wound within 30 days following three topical applications of rose stem cell-derived exosomes (269). The same group also reported favorable therapeutic outcomes of rose stem cell-derived exosomes in ischemic ear flap wounds (270). In another patient presenting with an acneiform rash and pruritus, inflammation largely subsided within 72 hours after treatment with rose stem cell-derived exosomes, and the skin appearance returned to normal (271). Rose stem cell-derived exosomes have also been shown to hold therapeutic value in skin treatment following radiofrequency microneedling (272). Related literature has reported eight clinical cases further demonstrating the therapeutic effects of rose stem cell-derived exosomes in a variety of skin conditions, including atopic dermatitis, post-skin-graft scarring, scald burns, sunburn, and hyperpigmentation (273).

These clinical findings suggest that therapies based on MSC-Exos and plant-derived exosomes hold therapeutic potential and acceptable short-term safety across a broad spectrum of skin diseases, and more exosome-based therapeutic strategies are likely to be applied to the treatment of human skin disorders in the future.

At present, although exosomes have demonstrated some visible efficacy in clinical trials of wound healing, several issues must be addressed before exosome-based therapies can be translated into clinical practice. Firstly, a primary obstacle is the lack of unified standards for exosome isolation and purification. Current techniques, including ultracentrifugation, density gradient centrifugation, ultrafiltration, and immunoaffinity capture, each present distinct trade-offs in purity, yield, scalability, and biomolecular preservation. The absence of a standardized workflow results in considerable batch-to-batch variability, complicating the assurance of consistent product quality and bioactivity across studies. Moreover, existing methods are often inadequate for effectively separating exosomes from other extracellular vesicle subtypes. An ideal isolation protocol that simultaneously ensures high purity, integrity, functional preservation, and scalability remains to be established. Although studies suggest that storage at -20 °C, -80 °C, or in lyophilized form can preserve exosome activity for several weeks (98), systematic norms for long-term storage, transportation, and functional characterization are still lacking.

Secondly, there is a critical need for standardized frameworks to optimize exosome activity and ensure rigorous quality control, which is essential for guaranteeing clinical safety and efficacy (274). Exosome quality is influenced by numerous variables, including donor cell source, culture conditions, passage number, and isolation methodology. The development of universally accepted quality standards and robust analytical methods, including encompassing identity, purity, potency, and stability, is urgently required. The starting material is equally crucial; the use of well-characterized, high-quality MSCs under optimized culture conditions is fundamental to obtaining exosome preparations with consistent therapeutic potency.

Finally, although numerous *in vitro* and preclinical animal studies support the therapeutic potential of MSC-Exos and plant-derived exosomes, clinical evidence remains relatively limited. Animal models cannot fully replicate the complexity and heterogeneity of human wound pathophysiology. Furthermore, substantial variations in reported exosome dosage, administration routes, and treatment regimens across studies hinder effective comparison and meta-analysis, thus providing limited actionable guidance for clinical trial design.

In conclusion, advancing exosome-based therapies into clinical reality requires concerted efforts across multiple fronts. Mechanistically, deeper investigation is needed to decipher the synergistic actions of exosomal cargo and their precise interactions with target cells in the wound microenvironment. Technologically, the development of efficient, scalable, and standardized methods for exosome production, purification, and quality control is paramount to ensure product safety and consistency. Clinically, more rigorously designed, large-scale trials are essential to validate the efficacy of stem cell exosomes across diverse wound types and patient populations. Addressing these challenges will accelerate the transition of exosome therapies from promising laboratory research to reliable clinical treatments, ultimately offering improved outcomes for patients with acute and chronic skin wounds.

## 7 Discussion

Cutaneous wound healing is a highly coordinated yet vulnerable biological process. Dysregulation of immune responses, persistent inflammation, and disruption of the skin barrier collectively contribute to homeostatic imbalance and impaired tissue function (275). This is particularly evident in chronic wounds, such as diabetic foot ulcers, where the healing process often stagnates in the inflammatory phase, failing to progress effectively to proliferation and remodeling (13). While conventional therapies can provide a passively favorable microenvironment, they are generally insufficient to actively correct underlying cellular and molecular dysfunction (276). Against this background, MSC-Exos have emerged as a promising cell-free therapeutic strategy in skin regenerative medicine, although their clinical translation remains in its early stages.

As natural nanocarriers, MSC-Exos are capable of delivering complex bioactive cargo, including proteins, lipids, and nucleic acids, to recipient cells within the wound bed (277), thereby partially replicating the regenerative functions of their parent stem cells while circumventing issues associated with cell transplantation, such as immune rejection, tumorigenicity, and ethical concerns (278). The activity of stem cell-derived exosomes does not operate through isolated biological effects, but rather maps onto the sequential phases of wound healing: during the inflammatory phase, they modulate immune cell behavior and promote macrophage polarization toward a reparative phenotype; during the proliferative phase, they stimulate the proliferation and migration of fibroblasts and keratinocytes and enhance angiogenesis; and during the remodeling phase, they regulate collagen deposition and ECM remodeling to limit scar formation (279). By acting across these consecutive and overlapping phases, exosomes may recalibrate the entire healing cascade, constituting a multi-target therapeutic strategy that is currently supported primarily by preclinical evidence.

Among exosomes derived from different tissue sources, ADSC-Exos have attracted considerable attention owing to their abundant source and ease of acquisition; BMSC-Exos represent the most extensively investigated type, supported by a broad preclinical evidence base; hUC-Exos offer potential translational advantages attributable to their low immunogenicity and robust proliferative capacity; AnSC-Exos, rooted in the unique context of complete organ regeneration in deer antlers, open new avenues for scarless wound healing research; USC-Exos are characterized by a simple isolation process, relatively low cost, and the absence of invasive procedures, whereas iPSC-Exos exhibit exceptional differentiation potential. Meanwhile, PELNs from sources such as aloe, ginseng, and ginger, leveraging their abundant availability, low immunogenicity, controllable cost, and natural enrichment in antioxidants, have shown promise in mitigating oxidative stress and inflammation (280, 281). Although PELNs remain less mature than MSC-Exos in terms of mechanistic understanding and translational evidence, they may serve as a complementary and alternative source, particularly in resource-limited settings or where concerns arise regarding stem cell sourcing.

Engineering strategies may further broaden the application scope of exosome-based therapies. Exosomes can be engineered to carry defined therapeutic cargo, such as specific miRNAs, proteins, or small-molecule drugs, through genetic modification of donor cells or direct cargo loading, thereby enabling targeted modulation of dysregulated pathways in refractory wounds (282). Incorporating exosomes into functionalized biomaterials, such as thermosensitive hydrogels and chitosan-based liquid bandages, can further achieve sustained release and improved local retention at the wound site (283, 284). Looking ahead, surface modification for cell-specific targeting, combined with scalable and quality-controlled production, may support the development of indication-specific exosome formulations tailored to different wound types and healing phases, although most of these engineering approaches remain at the preclinical proof-of-concept stage.

Critically, the existing clinical evidence must be clearly distinguished from the broader and more promising body of preclinical findings. Most human studies reported to date are small-scale trials, case reports, or case series with short follow-up periods. Although encouraging signals have been reported, for instance, hUC-MSC-based interventions improved neovascularization and ulcer healing in diabetic foot ulcers (255), and ADSC-Exos combined with laser therapy achieved satisfactory outcomes in atrophic acne scars and skin aging (262) (263), these observations derive from limited sample sizes and heterogeneous protocols, and randomized controlled evidence confirming efficacy remains scarce. Data on long-term outcomes, including recurrence, delayed adverse events, and the durability of treatment benefits, are largely absent.

Several practical challenges also impede broad clinical translation. The lack of standardized, reproducible protocols for exosome isolation, purification, characterization, and storage leads to batch-to-batch variability and difficulties in clinical reproducibility (285, 286). Unlike well-defined conventional drugs, exosomes are complex natural products carrying heterogeneous cargo (77), which poses considerable challenges for quality control, potency assays, and pharmacokinetic evaluation. Moreover, substantial variation in dosing, administration routes, and treatment regimens across studies hampers effective comparison and analysis, offering limited guidance for clinical trial design. Future research should prioritize the following directions: First, establish standardized systems for the isolation, purification, and quality control of MSC-Exos to ensure batch-to-batch consistency and reproducibility. Second, deeply dissect the functional heterogeneity of MSC-Exos derived from different tissue sources to clarify their optimal indications and patient populations. Third, conduct more rigorously designed, adequately powered randomized controlled clinical trials to validate, with high-quality evidence, the therapeutic efficacy and long-term safety of MSC-Exos in acute and chronic skin wounds. Fourth, explore the deep integration of MSC-Exos with functionalized biomaterials and engineering modification technologies to further enhance their targeted delivery efficiency and therapeutic outcomes.

## 8 Conclusion

In summary, stem cell-derived exosomes act across the sequential phases of skin wound healing, including modulating inflammation, promoting cell proliferation and angiogenesis, and coordinating ECM remodeling, thus representing a promising multi-target, cell-free strategy, with PELNs serving as a potential complementary source. However, their clinical application remains in its infancy: current human evidence is largely limited to safety and feasibility signals from small-scale studies, and efficacy, long-term safety, and the durability of therapeutic benefit have yet to be established. Only through concerted advances in these aspects can exosome therapy be rigorously evaluated and, once validated, progressively translated from promising laboratory research into a dependable clinical treatment for patients with acute and chronic skin wounds.
